# Research data warehouse: using electronic health records to conduct population-based observational studies

**DOI:** 10.1093/jamiaopen/ooad039

**Published:** 2023-06-21

**Authors:** Wansu Chen, Fagen Xie, Don P Mccarthy, Kristi L Reynolds, Mingsum Lee, Karen J Coleman, Darios Getahun, Corinna Koebnick, Steve J Jacobsen

**Affiliations:** Department of Research and Evaluation, Kaiser Permanente Southern California, Pasadena, California, USA; Department of Research and Evaluation, Kaiser Permanente Southern California, Pasadena, California, USA; Department of Research and Evaluation, Kaiser Permanente Southern California, Pasadena, California, USA; Department of Research and Evaluation, Kaiser Permanente Southern California, Pasadena, California, USA; Department of Health Systems Science, Kaiser Permanente Bernard J. Tyson School of Medicine, Pasadena, California, USA; Department of Cardiology, Los Angeles Medical Center, Southern California Permanente Medical Group, Los Angeles, California, USA; Department of Research and Evaluation, Kaiser Permanente Southern California, Pasadena, California, USA; Department of Health Systems Science, Kaiser Permanente Bernard J. Tyson School of Medicine, Pasadena, California, USA; Department of Research and Evaluation, Kaiser Permanente Southern California, Pasadena, California, USA; Department of Health Systems Science, Kaiser Permanente Bernard J. Tyson School of Medicine, Pasadena, California, USA; Department of Research and Evaluation, Kaiser Permanente Southern California, Pasadena, California, USA; Department of Health Systems Science, Kaiser Permanente Bernard J. Tyson School of Medicine, Pasadena, California, USA; Department of Research and Evaluation, Kaiser Permanente Southern California, Pasadena, California, USA

**Keywords:** electronic health record, research data warehouse, health care utilization, data quality, integration, standardization, chronic disease prevalence

## Abstract

**Background:**

Electronic health records and many legacy systems contain rich longitudinal data that can be used for research; however, they typically are not readily available.

**Materials and methods:**

At Kaiser Permanente Southern California (KPSC), a research data warehouse (RDW) has been developed and maintained since the late 1990s and widely extended in 2006, aggregating and standardizing data collected from internal and a few external sources. This article provides a high-level overview of the RDW and discusses challenges common to data warehouses or repositories for research use. To demonstrate the application of the data, we report the volume, patient characteristics, and age-adjusted prevalence of selected medical conditions and utilization rates of selected medical procedures.

**Results:**

A total of 105 million person-years of health plan enrollment was recorded in the RDW between 1981 and 2018, with most healthcare utilization data available since early or middle 1990s. Among active enrollees on December 31, 2018, 15% were ≥65 years of age, 33.9% were non-Hispanic white, 43.3% Hispanic, 11.0% Asian, and 8.4% African American, and 34.4% of children (2–17 years old) and 72.1% of adults (≥18 years old) were overweight or obese. The age-adjusted prevalence of asthma, atrial fibrillation, diabetes mellitus, hypercholesteremia, and hypertension increased between 2001 and 2018. Hospitalization and Emergency Department (ED) visit rates appeared lower, and office visit rates seemed higher at KPSC compared to the reported US averages.

**Discussion and conclusion:**

Although the RDW is unique to KPSC, its methodologies and experience may provide useful insights for researchers of other healthcare systems worldwide in the era of big data analysis.

## INTRODUCTION

Although electronic systems have been implemented in large health organizations to support patient care, billing, and administration for decades, the Health Information Technology for Economic and Clinical Health (HITECH) Act enacted in 2009^1^ accelerated both the pace and the uniformity of data collection, thus making electronic health records (EHR) more attractive resources for observational research.^2–8^ Allowing researchers access to a large number of subjects with longitudinal health care records, so-called “big-data” can provide a quicker, more comprehensive, and cost-effective approach to access individual-level health care records. Notable EHR-based data sources include the Clinical Practice Research Datalink (CPRD), a primary care database that provides researchers around the world the opportunity to access National Health Service data from the UK,^9^ and the Veterans Administration’s Corporate Data Warehouse (CDW), a repository derived from the Veterans Health Administration’s electronic medical records system called Computerized Patient Record System (CPRS)/VistA system.^10^

Administrative data collected by healthcare organizations at the time of enrollment and during patient care are only sometimes readily available for research. First, the same data type (eg, hospital admission) may come from multiple data sources with various formats and inconsistent values. Second, the change of source data systems over time may leave the data fragmented. Third, the information collected administratively may be incomplete or contain duplicate entries. Fourth, data quality concerns critical to research usually need to be better documented than is possible with administrative data. Medical insurance claims data is a good example of electronic data that requires tremendous consolidation efforts. Although medical insurance data have been commonly used for research,^11^^,^^12^ the creation of encounters based on medical claims data has been a challenge,^12^ because the data are submitted for reimbursement purposes and thus do not represent the episode of care (eg, multiple claims for one hospital stay). Therefore, developing and maintaining a well-documented large-scale data infrastructure to support various research projects is crucial.

This report will describe the contents, development, maintenance methodology, and other aspects of a research data warehouse (RDW) within a large integrated healthcare system, Kaiser Permanente Southern California (KPSC). To demonstrate the application of the data in the RDW and the volume of data that can be used for various population-based research projects, we report descriptively the (1) total lengths of enrollment history, (2) demographic, behavioral, and other characteristics, (3) annual counts and age-adjusted prevalence of common chronic conditions, and (4) annual counts and rates of medical procedures performed in KPSC health plan enrollees.

## MATERIALS AND METHODS

### Population and environment

**Figure 1.**
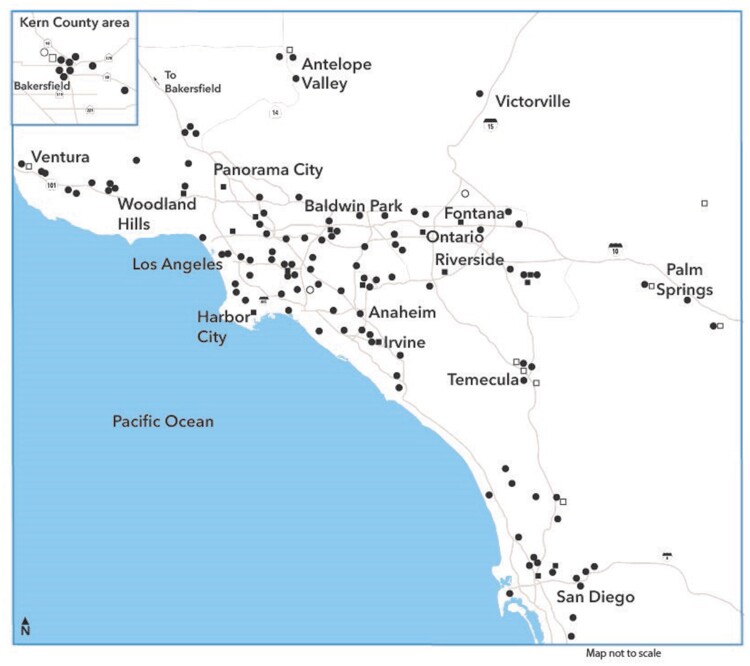
Geographic locations of KPSC medical centers and offices. Solid squares: KPSC hospitals; solid circles: KPSC medical offices; open squares: affiliated hospitals; open circles: affiliated medical offices.

Founded in 1945, KPSC provides integrated care to 4.8 million health plan enrollees at facilities throughout the Southern California region (Figure 1). At its 15 medical centers and 235 medical offices, some 7800 physicians and 25 000 nurses provide comprehensive care, including hospital admission, ambulatory care, ED, urgent care, office visits, optometry, rehabilitation, home health, and hospice care. Outside providers also render medical care for planned, referred, or emergency services. KPSC members are racially and ethnically diverse and represented 21% of the region’s population in 2020. The demographics and socio-economic status, including race and ethnic composition of the enrollees, are representative of those living in the region.^13^ This study was approved by the institutional review board of KPSC with a waiver for the requirement to obtain informed consent.

### HealthConnect

HealthConnect, customized and branded by Kaiser Permanente, is one of the largest civilian health care systems in the world. This comprehensive system replaced hundreds of legacy systems to integrate clinical records with appointments, registration (check-in), clinical documentation, orders, hospital admission/discharge/transfer/medication administration, and billing. In addition to the information that care providers directly enter, a large amount of data is loaded from other systems (eg, lab results) via an electronic interface. A unique medical record number is assigned at the time of health plan enrollment, and this number remains unchanged if an enrollee leaves and rejoins KPSC. In KPSC, the system was implemented between 2004 and 2008.

### Convergent medical terminology

With more than 75 000 concepts, convergent medical terminology (CMT) is a KP-wide solution to provide a consistent structure for medical terminology.^14^ The core of CMT comprises SNOMED-Clinical Terms (CT),^15–17^ Laboratory Logical Observation Identifiers Names and Codes (LOINC),^17–19^ and First Databank drug terminology.^20^^,^^21^ When a care provider diagnoses a patient’s medical conditions and enters them into KP HealthConnect, they are translated into CMT concepts. Most diagnosis-related concepts can be mapped to the International Classification of Diseases (ICD) codes in the backend database for the purpose of billing, reporting, and research. The same process occurs when a physician orders a medical procedure. A procedure-related concept (eg, lab, radiology) is mapped to a Current Procedural Terminology (CPT) code if such a mapping is feasible. Hospital-based services, including ED visits, are coded by professional coders based on international standard codes, and therefore, there is a lag of several weeks for hospital-based coding to be complete. For claims, the data are usually at least 90% complete within 3 months.

### Terminology standards

Terminology standards provide a foundation for clear interoperability and improved efficiency for information exchange. They are necessary for reproducibility and data consistency in collaborations across multi-organizations. A number of national/international and KPSC internal terminology standards are stored in the RDW (Table 1). Most of the KPSC internal codes can be mapped to national/international codes.

**Table 1.** National/international and KPSC internal terminology standards used in the RDW, by data mart

Data mart	Coding standards
Medical encounter	MS-DRG
Diagnosis	ICD-9-CM, ICD-10-CM, KPSC internal diagnosis codes
Procedure	ICD-9-CM, ICD-10-CM, CPT-4, HCPCS, KPSC internal procedure codes
Cause of death	ICD-9-CM, ICD-10-CM
Medication	NDC, Med-Span GPI, AHFS
Laboratory	CPT-4, LOINC, KPSC laboratory procedure codes
Immunization	CDC CVX codes
Pathology	ICD-9-CM, ICD-10-CM, SNOMED-CT
Radiology	CPT-4, CMT
Geocoding	FIPS/NIST
Problem list	ICD-9-CM, ICD-10-CM, CMT
Patient history	ICD-9-CM, ICD-10-CM, CPT-4, CMT
Provider	KPSC provider identifiers
Facility	KPSC facility identifiers

Medicare Severity Diagnosis Related Group (MS-DRG): https://www.cms.gov/Medicare/Medicare-Fee-for-Service-Payment/AcuteInpatientPPS/MS-DRG-Classifications-and-Software; ICD-9-CM/ICD-10-CM: International Classification of Diseases, Ninth/Tenth Revision, Clinical Modification (ICD-9-CM); CPT-4: Current Procedural Terminology; HCPCS: Healthcare Common Procedure Coding System (HCPCS); NDC: National Drug Code http://www.fda.gov/Drugs/DevelopmentApprovalProcess/UCM070829; GPI: Generic Product Identifier http://www.wolterskluwercdi.com/drug-data/why-medispan/; AHFS: American Hospital Formulary Service http://www.ahfsdruginformation.com/ahfs-pharmacologic-therapeutic-classification/; LOINC: logical Observation Identifiers Names and Codes; CVX: Vaccine Administered https://www2a.cdc.gov/vaccines/IIS/IISStandards/vaccines.asp?rpt=cvx; SNOMED-CT: Systematized Nomenclature of Medicine–Clinical Terms; FIPS: Federal Information Processing Standard https://www.nist.gov/itl/current-fips.

### Research data warehouse

The RDW was developed by integrating many data sources from the KPSC legacy and EHR systems as well as external data sources such as the American Community Survey into several different data marts (subsets of the RDW focusing on specific data content areas). The data update frequency varies by data content area with most patient care data updated on a weekly basis. The data content areas, development cycle, platform/storage, and data governance are described below.

#### Data content areas

**Figure 2.**
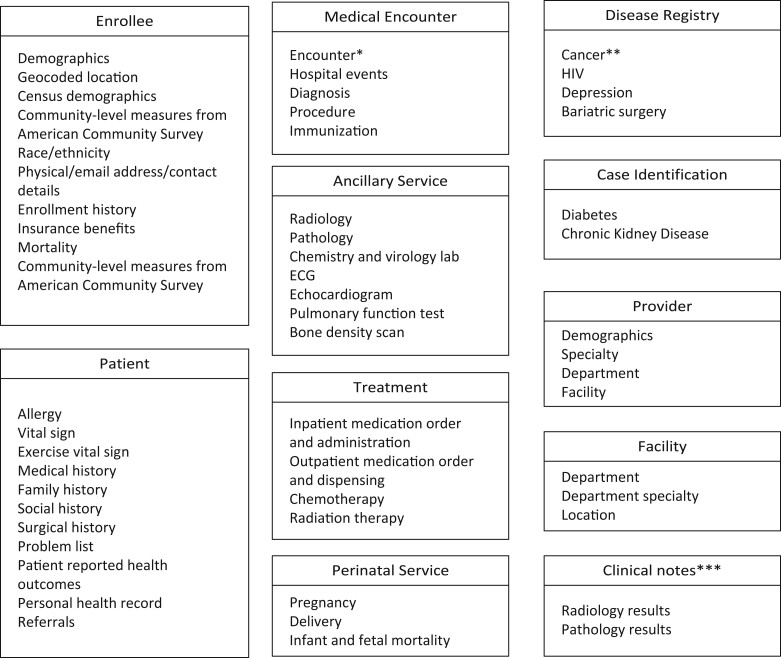
Common data marts. *Emergency department visits, inpatient stays, outpatient clinical visits (including urgent care and therapy), outside claims, home healthcare, skilled nursing facility use, and hospice care by both primary and specialty care providers. **Requested through KPSC Cancer Registry. ***Progress notes, ED provider notes, nursing notes, discharge summary notes, procedure notes and other types of notes are available from the reporting environment of HealthConnect.

The RDW contains a wide range of data content areas (domains). The most commonly used data content areas are shown in Figure 2. The RDW also contains radiology and pathology reports. At this point, digital videos/images in DICOM format and other types of notes, such as progress notes and discharge notes, are not included in RDW.

#### Development of business rules and data modeling

**Figure 3.**
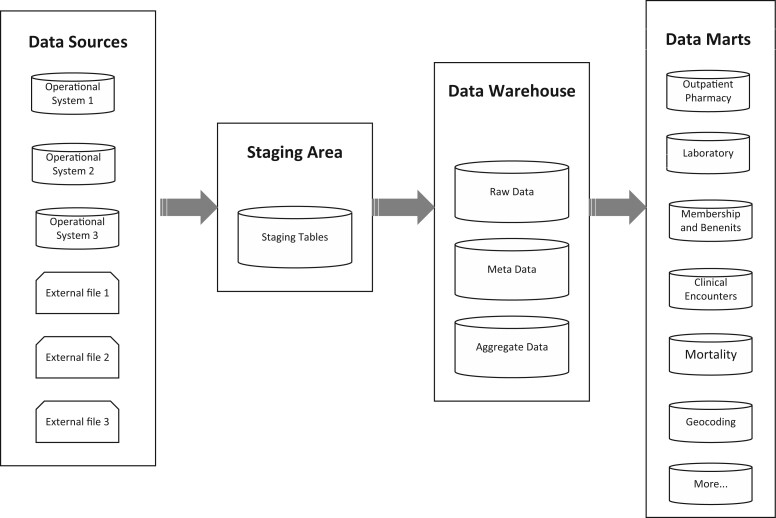
Data warehouse architecture.

Rules are defined to satisfy business needs. The extract, transform, load (ETL) process includes determining eligible data sources and records, data elements of interest, how these sources/records/data elements should be integrated and standardized, and how the final results are loaded into relational database tables (Figure 3). When data are extracted from multiple systems, the definitions, formats, and structures may vary; thus, the process is typically more complicated. In this step, the ETL developers also validate the rules using real data extracted from the sources. For illustration, we described (1) the rules of record consolidation of medical claims submitted by outside medical professionals to convert claims into episodes of care and (2) the rules of record cleaning/standardization of height and weight information in the vital signs data mart in Supplementary File S1.

#### ETL, integration testing, and analytical data quality check

After developing data models, the desired data are extracted, transformed, and loaded from the sources to a staging environment and subsequently to the target tables in a test or development environment according to the business rules and data models defined in the previous steps. Next, unit testing (to ensure that the ELT module correctly handles the target data) and integration testing (to ensure that all new and existing ETL components work together seamlessly) are performed. Test scenarios are prepared, and test cases are created. This testing includes the following tasks.

- Validation of counts between source data and final destination tables.
- Comparison of new data with current production data (if applicable).
- Validation of the existence of no unexpected duplication of data or orphaned records.
- Verification of accepted, default, invalid, or missing values.
- Verification of proper data types and formats.
- Validation of proper business logic implementation.
- Validation of the reasonable performance (in terms of time and of stress placed upon the network and on hardware) of the developed ETL module.
- Sample validation.

The ETL scripts are written by an ETL developer, reviewed by a second developer, and finally the data are reviewed by a quality assurance specialist before they are implemented to ensure that the scripts correctly reflect the specified requirements and perform appropriately.

For minor deployments, once the testing and quality assurance (QA) is complete, the module is deployed into production. This process involves the backup of existing tables. After the first run of the module, a quality assurance analyst then checks the data quality using the approach described in the Section “Data Quality Monitoring” (referred to as “analytical QA”). If an error is identified in the integration testing or analytical QA, the ETL scripts will be modified accordingly, and the code review, testing and analytical QA will be repeated.

With the release of the new databases/tables, a detailed documentation is published online within the KP network. The document includes at least the following sections: overview, data sources, data structures, sample program(s), data usage guideline, and potential data quality issues. For new development or major significant revisions of databases, user training is provided to inform users about the changes and their impact on research studies.

#### User acceptance testing, deployment, documentation, and user training

User acceptance testing (UAT) is performed for major deployments by the intended users/clients of the developed database or table. The UAT testers typically first identify test scenarios and create test cases based on business needs, then execute the test cases against the developed database, and document and share the test results with the developers. If any identified issues are caused by incorrect business rules or errors in ETL codes, the steps described in the previous 2 sections will be repeated. The success of UAT leads to the deployment of the data into the production environment.

#### Data quality monitoring

Monitoring data quality on a timely basis is an integral part of the RDW maintenance. There are a large number of automated scripts that provide email alerts to an on-call team of specialists that indicate whether a specific job has failed (and thus needs to be re-run), whether unexpected values for a particular data element have been detected (eg, a new CPT or LOINC value in the source data), or whether there are unexpected counts in the loaded data (eg, a decrease in counts). In addition, on a routine basis, QA reports are generated and posted to a shared location where users have access. The updated frequencies of the QA reports match the updated frequencies of the data. For example, weekly reports are generated for weekly updated data. These reports are presented in both table and graphic formats to allow for the examination of yearly, monthly, and weekly trends, as well as changes from previous versions of the data (Supplementary File S2).

#### Platforms and storage

The largest RDW tables are hosted in a high-performance Oracle Exadata environment which leverages a massively parallel grid architecture. This server is located within a KP secure data center. Smaller and less-used tables are currently hosted in an Oracle environment but are being migrated to the above-mentioned Exadata environment. ETL scripts run on a SAS Business Intelligence platform consists of 3 computational servers and 3 metadata servers within a grid environment. With the flexible data management technology, the SAS platform is also used for many data integration processes with the remainder being performed by PL/SQL packages running directly in the Exadata environment. The warehouse architecture and ETL designs were created to keep minimum data transfer across the KP network.

#### Scientific input

The Data Scientific Oversight Committee, representing diverse perspectives of scientists, statisticians, programmers, and project managers, provides scientific guidance and oversight on RDW development. More specifically, it evaluates proposals for new database/table development and provides recommendations on business rules of data contents and development priorities.

#### Data governance

The management of the RDW is governed by the existing policies and standard operation procedures within the Department of Research and Evaluation and those enforced by the Kaiser Permanente Information Technology and the Technology Risk Assessment Office. Only people who have a business justification for using the data have access. Sensitive data such as social security numbers are protected using Oracle’s Virtual Private Database feature, which are database policies that are automatically enforced by controlling access at the column or row level. Any requests to access sensitive data require 3 levels of approval. The first is by the requester’s manager, followed by the data owner, and finally by the data service manager. Due to patient confidentiality, direct access to the RDW is not granted to external researchers; however, access to the data may be permitted through collaboration with KPSC researchers.

#### Change management

Small changes to the data (eg, the introduction of a new data element) may result in a relatively minor hotfix to the ETL which is deployed quickly. Such minor changes are typically identified in one or more of 3 ways: bugs reported by users, automated monitoring alerts (eg, a new value of a key variable), or manually reviewed QA reports. Major changes to the data arising from a major change in the source data system (eg, the retirement of one data system and its replacement with another one) occur less frequently and result in a larger project which is then prioritized based on multiple factors including the urgency (eg, scheduled system change), the complexity of the work, and the available resources. The priorities are often discussed within the Data Scientific Oversight Committee. Addition of new functionalities also follows the same process.

Change requests to the database structure or scripts in production are evaluated by the Change Management Committee. The Committee reviews and approves the requests based on factors such as types of changes (ie, enhancement, defect, database/application, server updates), user impact, complexity of implementation plans including rollback plan in case of failure, outage requirement, priority, and potential risks.

#### Cost-saving statements

The RDW dramatically simplifies data extraction process. First, it is much more convenient to extract data elements from RDW than from original data sources. As an example, data elements commonly used for research are extracted from more than 150 tables in the source EHR and claims data warehouses, transformed by ETL scripts and loaded into a data mart in RDW with only 14 tables for users to use. Second, the RDW contains external data sources that are both challenging and time-consuming to extract. For example, death and cause of death are derived from vital statistics records through a complicated linkage process.^22^^,^^23^ Neighborhood level measures (eg, neighborhood deprivation index) are appended after health plan enrollees’ addresses are geocoded.^24^ Each linkage or geocoding process involves a degree of expert knowledge and takes some 2–3 weeks to perform. Finally, the RDW includes legacy data related to utilization (encounter, procedure, and diagnosis data), labs, pharmacy, radiology, and membership which are generally no longer available outside the RDW. As a concrete example to demonstrate cost saving, one might consider a typical research project that needs to make use of health care utilization, diagnosis, medical procedures, vital signs, pharmacy, labs, membership, demographics, and neighborhood level data (eg, neighborhood deprivation index). Using the RDW, a user would only need to extract data from 12 database tables or views (a view is the result of a prewritten, stored query that are saved within the database users can query much as they would a table) in the RDW. However, if the user were to use the source data, they would need to query more than 200 tables from 3 different databases. He/she would also need to perform complex joins and transformations on these tables which the RDW ETL performs. In addition, he/she would need to link study subjects to vital statistics data as well as to geocode their addresses to enable the usage of community-level data. Conservatively, the process of extracting data from the sources would take at least 4 weeks assuming that the user is very familiar with the various source systems and the linkage process.

#### Number of users

There are currently 114 active users of the RDW measured as individuals with accounts and 85 measured as users who have extracted data from the RDW over the last 12 months. In addition, there are hundreds of passive users who use a web-based application to access partial data in the RDW for the purpose of research feasibility assessment.

#### Allocation of resources

The allocation of efforts changes overtime as the business needs and the maturity of the RDW increase. On an average, more than half of the efforts are spent on development/enhancement and implementation of new database tables and/or new data elements development, and about 10–20% of efforts are used for maintenance which includes ETL and data quality monitoring.

### Application

To demonstrate the volume and the application of the integrated data in the RDW, we reported (1) the total person-years of health plan enrollment (ie, summation of enrollment years from all study subjects) in each of the 2-time windows (1981–2000, 2001–2018), (2) demographic and clinical characteristics of active enrollees on December 31, 2018, (3) total counts, crude and age-adjusted annual prevalence of selected common chronic conditions in 2001–2018, and (4) total counts, crude and age-adjusted annual rate of selected medical utilization including common medical procedures in 2001–2018. The age adjustment was made using the direct method with the 2000 US Census population as the standard population.^25^^,^^26^ Enrollees who were not enrolled in the KPSC health plan for at least 11 months within each calendar year were excluded from the analyses of (3) and (4) above. The race/ethnicity information is derived from multiple data sources based on a sophisticated algorithm (including imputation). The information is mainly self-reported since 2009, and the data sources were previously described.^27^ The information on smoking status (current, passive, quit, and never) is collected during clinic visits. The descriptions of the selected chronic conditions are listed in the footnote of Table 4 and Supplementary File S3. Diabetes, heart failure, and hypertension were each defined using 2 different algorithms (Supplementary File S3) to demonstrate the impact of the variations on disease prevalence estimates.

## RESULTS

Between 1981 and 2018, there was a total of 105 million person-years (43 million in 1981–2000 and 62 million in 2001–2018) (Table 2). Of which, 27, 41, 25, and 11 million person-years were attributed to enrollees of 0–17, 18–44, 45–64, and ≥65 years of age, respectively. Non-Hispanic white members had the highest number of person-years of enrollment (35 million), followed by Hispanic (30 million), African Americans (10 million), and Asian (7 million) members. Between 2001 and 2018, Hispanic members contributed the highest number (22 million) of person-years among all the ethnic groups.

**Table 2.** Enrollee population in person-years, overall by age, sex, and race/ethnicity, 1981–2018

Demographics	Total person-years		
	1981–2000	2001–2018	Total
All	42 831 837	61 796 375	104 628 212
Age,^a^ years			
0–17	12 398 384	14 897 528	27 295 912
18–44	17 873 225	23 467 270	41 340 496
45–64	8 847 439	15 719 290	24 566 730
≥65	3 712 787	7 712 286	11 425 073
Sex			
Male	20 744 845	29 942 760	50 687 606
Female	22 086 288	31 852 051	53 938 339
Other	702	1562	2264
Race/ethnicity			
Non-Hispanic white	14 336 572	20 652 583	34 989 156
Hispanic	7 247 682	22 974 254	30 221 936
African American	4 252 865	5 605 909	9 858 774
Asian	1 852 194	5 281 762	7 133 956
Pacific Islander	132 953	421 170	554 124
Native American	52 242	122 717	174 960
Multiple	54 704	220 759	275 464
Other	277 038	625 760	902 799
Unknown	14 625 583	5 891 455	20 517 038

a Age at the enrollee’s birthdate of each calendar year.

Among 4.4 million active enrollees on 12/31/2018, the median age was 38 years (IQR = 21, 57), and 48.5% were males with a median enrollment length of 5.2 years (IQR = 2.1, 12.8) (Table 3). Of the enrollees with known race/ethnicity, 33.9% were non-Hispanic white, 43.3% Hispanic, 8.4% African American and 11.8% Asian, or Pacific Islander. Of the active enrollees, 13.7% were insured under Medicare and Medi-CAL or other State programs covering 10.0%. Current and past smokers accounted for 4.7% and 16.1% of active enrolled adults (≥18 years of age) and 34.4% of children 2–17 years of age and 72.1% of adults ≥18 years of age were overweight or obese.

**Table 3.** Demographic and clinical characteristics of active enrollees on December 31, 2018

Demographic and clinical characteristics	Enrollees
All	4 393 380
Age,^a^ years	
Median	38
IQR [Q1, Q3]	[21, 57]
0–17	909 323 (20.7%)
18–44	1 720 321 (39.2%)
45–64	1 102 697 (25.1%)
≥65	661 039 (15.0%)
Sex	
Male	2 130 372 (48.5%)
Female	2 262 900 (51.5%)
Other	108 (0.0%)
Race/ethnicity	
Known	4 092 116 (93.1%)
Unknown	301 264 (6.9%)
Distribution among enrollees with known race/ethnicity	
Non-Hispanic White	1 386 913 (33.9%)
Hispanic	1 772 492 (43.3%)
American America	343 695 (8.4%)
Asian	450 889 (11.0%)
Pacific Islander	32 146 (0.8%)
Native American	9406 (0.2%)
Multiple/other	96 575 (2.4%)
Types of medical insurance^b^	
Commercial	3 147 548 (71.6%)
Medicare	599 667 (13.7%)
Medi-CAL or other State programs	440 925 (10.0%)
Individual	695 653 (15.8%)
Deductible HMO	834 355 (19.0%)
Duration of enrollment	
Median, year	5.2
IQR [Q1, Q3]	[2.1, 12.8]
Smoking status^c^	
Known	2 614 932 (75.1%)
Unknown	869 125 (24.9%)
Distribution among enrollees with known smoking status^c^	
Current	162 174 (6.2%)
Quit	561 522 (21.5%)
Passive	22 747 (0.9%)
Never	1 868 489 (71.4%)
Weight categories	
2–17 years of age	
Known	627 915 (76.0%)
Unknown	197 756 (24.0%)
Distribution among enrollees with known weight categories	
Underweight—BMI < 5th percentile	24 448 (3.9%)
Normal weight—BMI ≥5th and <85th percentile	387 354 (61.7%)
Overweight—BMI ≥85th and <95th percentile	99 632 (15.9%)
Obese—BMI ≥95th percentile	116 210 (18.5%)
Extreme obese—BMI ≥35 kg/m^2^ or weight >400 pound	271 (0.0%)
≥18 years of age	
Known	2 633 326 (75.6%)
Unknown	850 731 (24.4%)
Distribution among enrollees with known BMI categories	
Underweight—BMI <18	23 154 (0.9%)
Normal weight—BMI ≥18 and <25	714 251 (27.1%)
Overweight—BMI ≥25 and <30	886 791 (33.7%)
Obese class I—BMI ≥30 and <35	567 910 (21.6%)
Obese class II—BMI ≥35 and <40	263 784 (10.0%)
Obese class III—BMI ≥40 and <50	151 714 (5.8%)
Obese class IV—BMI ≥50	25 722 (1.0%)

a Age on December 31, 2018.

b Not mutually exclusive.

c Limited to adults ≥18 years of age.

The age-adjusted prevalence of the selected medical conditions are shown in Table 4. Asthma prevalence doubled in the period of 18 years (3.2% in 2001 and 6.5% in 2018). Atrial fibrillation prevalence steadily rose in people ≥60 years of age, ranging from 3.9% in 2001 to 6.4% in 2018. COPD prevalence increased from 1.8% in 2001 to 2.2% in the late 2000s and decreased to 1.6% in 2017–2018. Prevalence of diabetes mellitus based on both definitions were about 6% in 2001 and climbed to 11–12% in 2016–2018. Hypercholesteremia was less frequent (8.8–16.6%) prior to 2007 and remained in the range of 21–23% between 2007 and 2018. Hypertension based on definition 1 (only reported for 2008–2018) identified about 40% of adults as having hypertension, while the prevalence based on definition 2 identified half or less during the same time period. Nearly a third of the adult population (30–32%) were obese (only reported for 2008–2018). Heart failure prevalence based on both definitions stayed flat during the study period. The counts and the unadjusted prevalence of these medical conditions can be found in Supplementary File S4.

**Table 4.** Age-adjusted annual prevalence (%) of selected chronic conditions and annual rates (/person or 1000 persons) of medical utilization in adults aged ≥18 years,^a^ 2001–2018

	2001	2002	2003	2004	2005	2006	2007	2008	2009	2010	2011	2012	2013	2014	2015	2016	2017	2018
**Medical conditions**																		
Asthma	3.2	3.3	3.5	3.5	3.9	4.0	4.8	5.0	5.4	5.3	5.3	5.3	5.4	5.7	6.0	6.2	6.5	6.5
Atrial fibrillation (≥60 yrs of age)	3.9	4.0	4.7	4.9	5.1	5.2	5.5	5.5	5.6	5.7	5.8	5.9	6.0	6.1	6.1	6.2	6.3	6.4
COPD	1.8	1.7	1.9	2.0	2.1	2.1	2.2	2.2	2.2	2.1	2.0	1.9	1.8	1.8	1.8	1.7	1.6	1.6
Diabetes mellitus (definition 1)	6.4	6.6	7.0	7.4	7.9	8.4	8.9	9.1	9.4	9.7	9.9	10.2	10.2	10.9	11.2	11.6	11.9	11.8
Diabetes mellitus (definition 2)	5.7	6.0	6.4	6.8	7.2	7.7	8.3	8.6	9.0	9.2	9.4	9.5	9.6	9.9	10.1	10.5	10.7	10.5
Heart Failure (definition 1)	1.4	1.3	1.5	1.6	1.7	1.7	1.7	1.6	1.6	1.5	1.5	1.4	1.4	1.4	1.4	1.4	1.5	1.6
Heart Failure (definition 2)	0.5	0.5	0.5	0.6	0.6	0.6	0.7	0.7	0.7	0.7	0.6	0.6	0.6	0.6	0.6	0.6	0.5	0.5
Hypercholesteremia	8.8	10.4	12.3	14.2	15.4	16.6	21.4	22.0	22.5	22.9	22.7	22.1	22.2	22.0	21.6	21.5	21.5	21.5
Hypertension (definition #1)^b^								41.2	41.4	40.9	40.4	40.3	40.3	40.1	39.8	40.0	39.7	39.4
Hypertension (definition #2)^b^	12.2	12.6	13.6	15.9	17.7	19.9	21.4	21.6	21.9	21.7	21.1	20.5	20.2	19.8	18.9	18.4	17.7	17.6
Obesity^b^								30.1	31.0	31.0	31.1	31.0	31.0	31.5	31.8	32.3	32.3	32.4
**Medical utilization**																		
Hospital admission/kp	99.72	99.86	99.77	113.11	114.02	103.58	100.07	102.14	98.81	96.93	91.66	85.45	81.65	77.63	75.35	70.04	66.25	64.21
ER visits/kp	376.26	331.06	305.18	293.37	284.86	264.16	261.56	260.68	272.30	262.76	263.36	267.47	259.70	257.32	261.43	259.85	263.07	261.55
Office visits/p	6.24	6.16	6.31	6.59	6.86	6.51	6.07	5.73	5.94	5.90	5.89	5.92	5.91	5.82	5.68	5.65	5.44	5.47
Lab results^c^/p	38.78	39.45	39.78	40.32	40.31	41.53	40.99	39.86	41.00	41.59	41.96	41.55	41.69	40.24	38.97	39.53	39.78	40.13
Outpatient med dispense/p	8.70	8.56	8.68	8.46	8.74	8.81	8.83	9.03	9.16	9.13	9.06	9.05	9.03	8.99	9.02	9.04	8.97	8.83
Blood pressure assessment^b^/p								4.56	4.93	4.93	4.85	4.80	4.71	4.64	4.53	4.47	4.36	4.32
Body weight assessment^b^/p								3.41	3.79	3.83	3.90	3.91	3.82	3.85	3.80	3.74	3.61	3.56
Prophylactic vaccine/kp	308.27	325.48	396.58	363.17	401.24	377.67	436.62	453.81	550.43	619.59	697.77	700.97	684.57	716.98	755.18	718.31	729.63	774.97
Delivery^d^/kp	50.84	49.61	50.17	49.78	49.86	50.75	50.84	50.84	49.42	49.84	49.65	50.99	50.67	50.21	49.27	48.32	45.99	45.30
EDG/kp	13.94	14.24	14.09	13.17	13.16	12.86	12.95	18.60	19.66	20.18	19.71	20.26	19.81	19.94	19.75	21.52	21.61	21.76
Colonoscopy^e^/kp	31.09	35.07	39.05	38.13	48.63	58.82	67.16	92.41	97.53	99.20	98.37	103.85	102.31	97.85	92.71	94.72	96.60	99.55
Sigmoidoscopy^e^/kp	59.02	44.55	39.85	38.48	40.19	32.70	30.62	29.40	26.30	30.58	21.15	15.73	10.68	7.57	4.89	3.87	3.19	2.69
FOBT/FIT^e^/kp	86.71	91.21	92.31	92.09	83.33	148.47	264.81	312.74	330.33	332.13	339.52	349.33	364.23	353.18	360.76	344.31	331.55	332.37
Cardiac catheterization/coronary arteriography/kp	3.50	5.64	5.94	5.65	5.71	4.73	3.25	3.81	3.95	4.56	4.31	4.03	4.31	4.56	4.80	4.82	4.06	3.35
Electrocardiography/kp	186.61	201.93	206.90	227.83	234.82	233.56	221.12	223.18	230.50	234.40	225.95	222.79	210.76	203.65	204.70	211.10	207.03	215.12
Pulmonary function test/kp	27.65	37.76	29.18	24.30	20.18	20.78	14.93	19.85	40.96	38.00	39.81	37.41	37.42	34.47	32.16	31.11	30.08	29.59
CT scan^b^/kp		6.18	29.11	57.89	89.96	101.55	111.74	119.57	126.58	127.07	130.56	130.27	127.21	128.32	132.06	135.54	143.92	152.17
MRI scan^b^/kp		2.96	11.27	27.05	39.86	45.18	48.17	52.65	55.57	57.03	58.70	59.32	60.25	62.33	66.75	67.51	69.26	71.51
Ultrasound^b^/kp		9.21	32.25	59.99	90.91	112.54	131.79	137.65	148.06	160.07	166.45	173.03	177.30	179.93	179.12	178.24	179.39	183.67

Asthma: ICD-9: 493; ICD-10: J45.

Atrial fibrillation in patients ≥60 years of age: ICD-9: 427.31; ICD-10: I48.0, I48.1, I48.2, I48.91.

Chronic Obstructive Pulmonary Disease (COPD): ICD-9: 491.0, 491.1, 491.20, 491.21, 491.22, 491.8, 491.9, 492.0, 492.8, 493.20, 493.21, 493.22, 496; ICD-10: J41.0, J41.1, J41.8, J42, J43.0, J43.1, J43.2, J43.8, J43.9, J44.0, J44.1, J44.9.

Diabetes mellitus (definition 1): ICD-9: 250; ICD-10: E08-E13.

Diabetes mellitus (definition 2): ≥1 hospitalization with a principal discharge diagnosis or ≥ 2 outpatient encounter diagnoses or ≥ 1 prescription dispensed for an anti-diabetes medication. Exclusion criteria: (1) Patient diagnosed with gestational diabetes within 8 months from inpatient or outpatient diagnoses of diabetes (ICD-9 codes for gestational diabetes: 648.80, 648.81, 648.82, 648.83, 648.84, 790.21, 790.22, 790.29) AND/OR (2) Women only receiving Metformin or Thiazolidinedione with no inpatient or outpatient diagnosis of diabetes within 2 years.

Heart failure (definition 1): ICD-9: 428; ICD-10: I50.

Heart failure (definition 2): ≥ 1 hospitalization with a principal discharge diagnosis or ≥3 outpatient diagnoses of heart failure with (ICD-9: 398.91, 402.x1, 404.x1, 404.x3, 428.x; ICD-10: I50.x, I11.0, I13.0, I13.2, I97.130, I97.131, I09.81).

Hypercholesterolemia: Serum total cholesterol ≥ 240 mg/dl or taking cholesterol-lowering medication.

Hypertension (definition 1): ≥2 consecutive elevated BPs at nonurgent ambulatory visits on separate dates: SBP ≥130 or DBP ≥90 mmHg or taking antihypertensive medication.

Hypertension (definition 2): ≥2 outpatient diagnoses (ICD-9: 401–404, ICD-10: I10–I13) or ≥1 outpatient diagnosis of hypertension plus ≥1 anti-hypertensive drug prescription dispense(s) within (±) 1 year of the outpatient diagnosis.

Obesity in ≥20 years old: body mass index ≥30 kg/m^2^.

a Limited to adults who enrolled in the health plan for at least 11 months within the calendar year.

b Information not available or incomplete in early years.

c Chemistry and virology labs;.

d Women 15–44 years of age;.

e 50–75 years of age; EDG: esophagogastroduodenoscopy, also known as upper gastrointestinal endoscopy; FOBT: fecal occult blood test; FIT: fecal immunochemical test; /p: per person; /kp: per 1000 persons; med: medication.

The age-adjusted annual medical utilization rates, including common medical/surgical procedures, are reported in Table 4. During the study period, the age-adjusted annual hospital admission, ED visit, and office visit rates decreased by 36% (from 99.7 to 64.2/1000 adult enrollees), 30% (from 376.3 to 261.6/1000 adult enrollees), and 13% (from 6.3 to 5.5/adult enrollees), respectively (Table 4). The age-adjusted rates of prophylactic vaccine administration increased by 151%. The age-adjusted rates of esophagogastroduodenoscopy (EDG), colonoscopy and fecal occult blood test (FOT)/fecal immunochemical test (FIT) increased until 2008–2009 and have remained unchanged since then. The raw counts and the unadjusted utilization rates can be found in Supplementary File S5. In 2018, there were 207.8k hospital admissions, 0.84 million ED visits, 18.0 million outpatient office visits, 21.9 million outpatient medication dispensing, 132.8 records of chemistry and virology laboratory test results, 14.1 million blood pressure measurements, and 11.6 million weight measures in adult patients aged ≥18 years (Supplementary File S5).

## DISCUSSION

We created an RDW with integrated information from multiple internal and external data sources. With a total of 105 million person-years of health plan enrollment between 1981 and 2018, the RDW provides individually linked and source-integrated administrative and clinical data for researchers to conduct population-based studies. The integrated and standardized data have offered convenience and consistency to thousands of research projects conducted at KPSC since it was first established in the late 1990s. Majority of the studies published by KPSC researchers (eg, more than 650 publications in peer-reviewed journals in 2022) are supported by the RDW.

Data in the RDW serve multiple types of research projects. For epidemiological studies, health plan enrollment information in the RDW provides denominators to estimate prevalence and incidence of health conditions, risk factors, and diseases. Retrospective cohorts based on predefined inclusion/exclusion criteria can be assembled to evaluate the natural history of disease including treatments and responses through passive follow-up. Patients with prespecified diseases or exposure can be identified for screening and intervention with prospective monitoring. For health outcomes studies, the information can be used to understand practice patterns, define quality measures/outcomes, and evaluate the success of implemented interventions.^28^ For clinical informaticists, longitudinal and high-dimensional data can be utilized to develop and validate risk prediction models. For trialists, it is possible that the near real-time data may help identify potentially eligible study participants; however, additional filtering by manual chart review is often needed.

Compared to many large-size integrated clinical data warehouses or repositories within the United States previously reported such as Mayo Enterprise Data Trust developed by Mayo Clinic^29^ and the Synthetic Derivative developed by Vanderbilt University Medical Center,^30^ the RDW at KPSC is larger in size. The Veterans Health Administration’ CDW contains an impressive 9 million Veterans and covers multiple geographic areas^10^; however, the enrollees do not represent the United States general population because most of them are males, married and non-Hispanic white.^31^ Gagalova et al^32^ reviewed and compared more than 20 clinical research data warehouses with various sizes, data sources, and architecture models. Some of them contain data types that are not offered by the RDW. For example, the KPSC RDW does not include “omics” data and 2D or 3D images. Future clinical data warehouses may consider these types of data as the expenses for storage decrease.

The low prevalence of certain chronic conditions prior to 2007 could be due to under-coding of these conditions before the EHR was fully implemented at KPSC. Since the EHR was implemented (phased in over a period of 4 years), physicians are required to provide diagnosis codes before they can sign off medication orders, thus resulting in more complete coding of medical conditions. The majority of the age-adjusted prevalence rates of chronic conditions estimated in the current study appeared to be lower compared to those of California residents based on surveys.^33–37^ For example, the percent of adults who have been told they currently have asthma was in the range of 7.7–8.8% in California in 2011–2018, according to the Behavioral Risk Factor Surveillance System survey.^33^ This could be explained, at least partially, by the fact that self-reported health indicators, such as those derived from the Behavioral Risk Factor Surveillance System and the National Health Interview Survey, tend to be more complete compared to those that are derived from physician diagnoses, because not all patients seek medical care on an annual basis.

The current study defined 3 chronic conditions using different algorithms. For diabetes mellitus, the 2 algorithms resulted in comparable rates; however, the 2 algorithms of heart failure and hypertension yielded substantial differences (eg, rates based on definition 1 being 2 or 3 times larger than those of definition 2). The decision on which algorithm to choose is study specific. One may apply the more specific algorithm (eg, definition 2 for heart failure or hypertension) if the goal is to identify a group of patients with high positive predictive value.

According to the NHIS, 5.8%, 7.7%, and 16.7% of adults aged 18–44, 45–64, and ≥65 had overnight hospital stays in 2018.^38^ During the same year, the rate was 6.4 hospital admissions/100 adult enrollees (including repeated admissions) at KPSC, which appeared to be lower compared to the national average. The numbers of ER visits per 100 persons in the United States were 47.8, 45.4, 35.7, 45.2 per 100 persons in 2017 in residents 15–24, 25–44, 45–64, and ≥65 years of age,^39^ compared to the age-adjusted rate of 26.3/100 adult enrollees at KPSC. The lower rates of hospital admission and ER visits at KPSC compared to the national averages could be due to the more frequent medical care in clinic settings (5.7 visits/adult person at KPSC vs. 1.5, 2.1, 3.0, and 5.0 visits/person in US residents 15–24, 25–44, 45–64 and ≥65 years of age in 2016).^40^

Given the large volume of data, data quality assessment is typically conducted by researchers specialized in specific medical area(s) with data elements commonly used for the research of these medical fields. For example, the accuracy of reporting maternal and fetal clinical diagnosis and procedural coding was validated by a team of perinatology researchers at KPSC.^41^ Data quality assessment domains and the assessment approach for each domain are described by Feder^42^ and Weiskopf et al^8^ A pragmatic framework was proposed to assess data quality in EHR-based clinical research for both single and multi-site studies.^43^ Data quality frameworks have been developed to clean data extracted from the EHR.^44^ Efforts are needed within each research project to validate and clean the project-specific data and to report any relevant quality issues.

There are apparent challenges when EHR data are being used for research. Because EHR data are not collected specifically to support research, they may bear the following weaknesses pertaining to data quality. First, coding for certain diseases provided by physicians may be incomplete or inaccurate.^45–47^ The level of incompleteness increases if the codes are not used for billing purposes. The problem is more severe for diseases defined by signs and symptoms. In 2 studies conducted at KPSC, Zheng et al^48^ and Yu et al^49^ revealed that using ICD-based codes alone to identify local reaction and anaphylaxis in vaccine safety studies could result in incomplete results because approximately 47% and 50% additional local reaction and anaphylaxis cases, respectively, can be identified at KPSC, when natural language processing of clinical notes was applied. In addition, the documentation in clinical notes could also be incomplete and inaccurate.^50^ Second, certain data terminologies and codes in EHRs lack standardization and, thus, cannot be used conveniently for research. The development of the RDW is a process of standardization in which the codes/values from multiple data sources are consolidated based on the clinical meaning of the codes/values. Pathak et al provided a framework in the SHARPn project to transform heterogeneous structured and unstructured data (eg, clinical notes) into a uniform and standardized infrastructure.^51^^,^^52^ More standards are expected to be implemented in commercial EHR software in the future. Third, a lack of consistent standardization across different organizations could occur due to different practice patterns and different terminology standards for services or products (even for the same EHR system/module). To provide a shared data structure to support multi-site studies, various types of research networks have implemented common data models.^53–56^ The adoption of common data models has advantages. For example, they provide fast access, large statistical power, and transparent duplication of analyses in multiple databases. However, information mapping from the source databases to the common data models could be incomplete.^57^ Multi-site validation is encouraged to improve the algorithm accuracy, as demonstrated in the eMERGE network in which thirteen EHR-based phenotype algorithms, including dementia, type 2 diabetes, and height were created and validated.^58^

In addition to the challenges mentioned above, the KPSC RDW has several limitations. First, the data in the warehouse is not real-time, thus studies that require real-time data need to extract the data directly from the original sources. Second, the availability of historical data depends on when the legacy systems were implemented. Third, the quality of the data varies depending on the clinical workflow and specialty area. For example, the slight decline in the prevalence of hypertension by definition 2 (defined by encounter diagnosis codes and hypertensive medications) could be due to the decrease in coding in recent years.

Despite the limitations mentioned above, the RDW at KPSC is advantageous compared to data derived solely from medical claims, for several reasons. First, medical records are available to conduct chart review for data/algorithm validation. Second, upcoding, which occurs when more serious conditions are coded and submitted than actually observed, an issue known for medical claims data, is unlikely to happen within KPSC because the service is prepaid for the vast majority of the patients. Third, clinical guidelines are strictly followed by KPSC providers to make clinical decisions/recommendations due to decision support tools and built-in system controls. These national or internal guidelines are developed based on medical research and are evidence-based. Therefore, medical practice across KP hospitals and medical offices could be more consistent. Finally, physician- and facility-level data are available to assess the impact of these characteristics on the outcomes of interest in addition to patient-level characteristics.

## CONCLUSION

The RDW provides a useful resource for the conduct of observational research based on EHR data, making the clinically and administratively collected data readily available for researchers. More comprehensive data quality assessment tools can be implemented to provide high-quality data for various types of research. More validation is encouraged to demonstrate the validity and report potential quality issues.

## Supplementary Material

ooad039_Supplementary_Data

## Data Availability

The data underlying this article cannot be shared publicly due to the privacy of individuals that are included in the study.
