# Supplementary material for: Research data warehouse: using electronic health records to conduct population-based observational studies
Source: JAMIA Open. 2023 Jun 21;6(2):ooad039. doi: 10.1093/jamiaopen/ooad039 (PMC10284679; doi:10.1093/jamiaopen/ooad039)
Supplement: ooad039_Supplementary_Data [file ooad039_supplementary_data.zip › Supplemental File 3.docx]

**Supplemental File 3. Definition of medical conditions for adults ≥18 years of age unless otherwise stated in the table below.**

| Medical conditions | Definition |
| --- | --- |
| Asthma | ICD-9: 493; ICD-10: J45 |
| Atrial fibrillation in patients ≥60 years of age | ICD-9: 427.31; ICD-10: I48.0, I48.1, I48.2, I48.91 |
| COPD | ICD-9: 491.0, 491.1, 491.20, 491.21, 491.22, 491.8, 491.9, 492.0, 492.8, 493.20, 493.21, 493.22, 496; ICD-10: J41.0, J41.1, J41.8, J42, J43.0, J43.1, J43.2, J43.8, J43.9, J44.0, J44.1, J44.9 |
| Diabetes mellitus (definition 1) | ICD-9:250; ICD-10: E08-E13 |
| Diabetes mellitus (definition 2) | ≥1 hospitalization with a principal discharge diagnosis or ≥ 2 outpatient encounter diagnoses or ≥ 1 prescription dispensed for an anti-diabetes medication. Exclusion criteria: (1) Patient diagnosed with gestational diabetes within 8 months from inpatient or outpatient diagnoses of diabetes (ICD-9 codes for gestational diabetes: 648.80, 648.81, 648.82, 648.83, 648.84, 790.21, 790.22, 790.29) AND/OR (2) Women only receiving Metformin or Thiazolidinedione with no inpatient or outpatient diagnosis of diabetes within 2 years. |
| Heart failure (definition 1) | ICD-9: 428; ICD-10: I50 |
| Heart failure (definition 2) | ≥ 1 hospitalization with a principal discharge diagnosis or ≥3 outpatient diagnoses of heart failure ICD-9: 398.91, 402.x1, 404.x1, 404.x3, 428.x  ICD-10: I50.x, I11.0, I13.0, I13.2, I97.130, I97.131, I09.81 |
| Hypercholesterolemia | Serum total cholesterol ≥ 240 mg/dl or taking cholesterol-lowering medication |
| Hypertension (definition 1) | ≥2 consecutive elevated BPs at non-urgent ambulatory visits on separate dates: SBP >= 130 or DBP >=90 mmHg, or taking antihypertensive medication |
| Hypertension (definition 2) | ≥2 outpatient diagnoses (ICD-9: 401-404, ICD-10: I10-I13) or ≥1 outpatient diagnosis of hypertension plus ≥1 anti-hypertensive drug prescription dispense(s) within (±) one year of the outpatient diagnosis |
| Obesity in ≥20 years old | Body mass index ≥ 30 kg/m^2^ |
