# Supplementary material for: Research data warehouse: using electronic health records to conduct population-based observational studies
Source: JAMIA Open. 2023 Jun 21;6(2):ooad039. doi: 10.1093/jamiaopen/ooad039 (PMC10284679; doi:10.1093/jamiaopen/ooad039)
Supplement: ooad039_Supplementary_Data [file ooad039_supplementary_data.zip › Supplemental File 4.docx]

**Supplemental File 4. Total counts and crude annual prevalence of selective chronic conditions in adults (18+ years of age), 2001-2018.**

|  | 2001 | 2002 | 2003 | 2004 | 2005 | 2006 | 2007 | 2008 | 2009 | 2010 | 2011 | 2012 | 2013 | 2014 | 2015 | 2016 | 2017 | 2018 |
| --- | --- | --- | --- | --- | --- | --- | --- | --- | --- | --- | --- | --- | --- | --- | --- | --- | --- | --- |
| ***Total counts*** |  |  |  |  |  |  |  |  |  |  |  |  |  |  |  |  |  |  |
| Asthma | 62,053 | 65,596 | 70,751 | 70,834 | 78,940 | 84,488 | 102,609 | 109,411 | 118,982 | 120,707 | 127,917 | 132,912 | 137,957 | 150,755 | 168,453 | 184,518 | 203,232 | 211,845 |
| Atrial Fibrillation** | 17,487 | 18,951 | 22,768 | 24,453 | 26,447 | 27,821 | 29,744 | 30,774 | 33,057 | 35,525 | 38,115 | 41,052 | 43,974 | 47,556 | 51,008 | 54,526 | 58,078 | 62,083 |
| COPD | 32,287 | 32,179 | 36,559 | 37,903 | 41,848 | 43,277 | 46,931 | 46,886 | 48,464 | 48,165 | 48,202 | 48,270 | 49,019 | 51,090 | 52,830 | 54,211 | 54,758 | 56,980 |
| Diabetes Mellitus (def 1) | 127,055 | 138,143 | 148,258 | 156,189 | 172,473 | 189,703 | 204,607 | 213,310 | 227,670 | 241,651 | 258,739 | 274,841 | 285,567 | 316,394 | 346,210 | 380,535 | 409,350 | 418,141 |
| Diabetes Mellitus (def 2) | 114,693 | 126,371 | 136,941 | 145,071 | 159,209 | 175,269 | 191,706 | 203,057 | 216,811 | 229,688 | 244,626 | 257,750 | 268,487 | 28,7751 | 314,187 | 342,753 | 366,567 | 373,648 |
| Heart Failure (def 1) | 22,860 | 23,529 | 26,794 | 28,922 | 31,511 | 32,448 | 33,556 | 32,504 | 33,231 | 33,836 | 34,651 | 35,283 | 36,000 | 37,856 | 40,360 | 43,975 | 47,135 | 53,327 |
| Heart Failure (def 2) | 9,032 | 8,916 | 9,150 | 10,134 | 10,700 | 11,015 | 13,571 | 14,342 | 14,957 | 15,452 | 14,827 | 14,828 | 15,398 | 16,647 | 17,537 | 18,540 | 19,030 | 20,674 |
| Hypercholesteremia | 176,270 | 217,616 | 263,499 | 302,847 | 338,871 | 375,639 | 494,927 | 520,367 | 546,152 | 574,315 | 594,062 | 603,498 | 626,145 | 648,026 | 681,955 | 716,846 | 753,780 | 786,615 |
| Hypertension (def 1) | 398,038 | 434,149 | 472,139 | 501,901 | 560,654 | 654,623 | 822,008 | 941,809 | 965,280 | 977,522 | 1,012,760 | 1,046,463 | 1,077,119 | 1,107,729 | 1,176,061 | 1,246,424 | 1,296,672 | 1,336,647 |
| Hypertension (def 2) | 236,720 | 256,657 | 283,644 | 330,673 | 379,113 | 443,422 | 487,340 | 500,073 | 521,443 | 534,975 | 544,119 | 551,838 | 562,184 | 574,004 | 585,816 | 600,804 | 606,166 | 624,032 |
| Obesity*** |  |  |  |  |  |  |  | 654,343 | 682,874 | 693,853 | 728,952 | 750,135 | 766,465 | 799,799 | 863,099 | 929,618 | 978,197 | 1,018,703 |
|  | 2001 | 2002 | 2003 | 2004 | 2005 | 2006 | 2007 | 2008 | 2009 | 2010 | 2011 | 2012 | 2013 | 2014 | 2015 | 2016 | 2017 | 2018 |
| ***Crude prevalence**** |  |  |  |  |  |  |  |  |  |  |  |  |  |  |  |  |  |  |
| Asthma | 3.2 | 3.3 | 3.5 | 3.6 | 3.9 | 4.1 | 4.8 | 5.1 | 5.4 | 5.4 | 5.4 | 5.5 | 5.5 | 5.9 | 6.2 | 6.4 | 6.7 | 6.7 |
| Atrial Fibrillation** | 3.1 | 3.1 | 3.6 | 3.9 | 4.0 | 4.1 | 4.3 | 4.3 | 4.5 | 4.6 | 4.6 | 4.8 | 4.9 | 5.1 | 5.1 | 5.1 | 5.2 | 5.4 |
| COPD | 1.7 | 1.6 | 1.8 | 1.9 | 2.1 | 2.1 | 2.2 | 2.2 | 2.2 | 2.2 | 2.1 | 2.0 | 2.0 | 2.0 | 1.9 | 1.9 | 1.8 | 1.8 |
| Diabetes Mellitus (def 1) | 6.6 | 6.9 | 7.4 | 7.9 | 8.6 | 9.2 | 9.6 | 9.9 | 10.4 | 10.9 | 11.0 | 11.3 | 11.5 | 12.4 | 12.6 | 13.1 | 13.4 | 13.2 |
| Diabetes Mellitus (def 2) | 6.0 | 6.3 | 6.8 | 7.4 | 8.0 | 8.5 | 9.0 | 9.4 | 9.9 | 10.3 | 10.4 | 10.6 | 10.8 | 11.2 | 11.5 | 11.8 | 12.0 | 11.8 |
| Heart Failure (def 1) | 1.2 | 1.2 | 1.3 | 1.5 | 1.6 | 1.6 | 1.6 | 1.5 | 1.5 | 1.5 | 1.5 | 1.4 | 1.4 | 1.5 | 1.5 | 1.5 | 1.5 | 1.7 |
| Heart Failure (def 2) | 0.5 | 0.4 | 0.5 | 0.5 | 0.5 | 0.5 | 0.6 | 0.7 | 0.7 | 0.7 | 0.6 | 0.6 | 0.6 | 0.7 | 0.6 | 0.6 | 0.6 | 0.7 |
| Hypercholesteremia | 9.1 | 10.9 | 13.1 | 15.4 | 16.9 | 18.2 | 23.3 | 24.1 | 25.0 | 25.9 | 25.3 | 24.8 | 25.1 | 25.3 | 24.9 | 24.7 | 24.8 | 24.9 |
| Hypertension (def 1) |  |  |  |  |  |  |  | 43.6 | 44.1 | 44.0 | 43.1 | 43.0 | 43.3 | 43.3 | 43.0 | 43.0 | 42.6 | 42.3 |
| Hypertension (def 2) | 12.3 | 12.8 | 14.1 | 16.8 | 18.9 | 21.5 | 23.0 | 23.1 | 23.8 | 24.1 | 23.2 | 22.7 | 22.6 | 22.4 | 21.4 | 20.7 | 19.9 | 19.7 |
| Obesity*** |  |  |  |  |  |  |  | 30.3 | 31.2 | 31.3 | 31.0 | 30.8 | 30.8 | 31.3 | 31.5 | 32.0 | 32.1 | 32.2 |

*Crude prevalence was estimated in adults who enrolled in the health plan for at least 11 months within the calendar year. **Estimated in patients 60+ years of age. ***Information prior to 2008 is incomplete.
