# Supplementary material for: Research data warehouse: using electronic health records to conduct population-based observational studies
Source: JAMIA Open. 2023 Jun 21;6(2):ooad039. doi: 10.1093/jamiaopen/ooad039 (PMC10284679; doi:10.1093/jamiaopen/ooad039)
Supplement: ooad039_Supplementary_Data [file ooad039_supplementary_data.zip › Supplemental File 5.docx]

**Supplemental File 5. Total counts and crude annual rates of medical utilization in adults (18+ years of age), 2001-2018.**

|  | 2001 | 2002 | 2003 | 2004 | 2005 | 2006 | 2007 | 2008 | 2009 | 2010 | 2011 | 2012 | 2013 | 2014 | 2015 | 2016 | 2017 | 2018 |
| --- | --- | --- | --- | --- | --- | --- | --- | --- | --- | --- | --- | --- | --- | --- | --- | --- | --- | --- |
| ***Total counts*** |  |  |  |  |  |  |  |  |  |  |  |  |  |  |  |  |  |  |
| Hospital admission (k) | 180.83 | 189.79 | 191.46 | 214.81 | 222.65 | 208.31 | 206.49 | 215.07 | 213.31 | 214.80 | 214.37 | 207.78 | 205.08 | 202.20 | 209.92 | 206.81 | 205.96 | 207.82 |
| ER visits (m) | 0.70 | 0.64 | 0.59 | 0.56 | 0.56 | 0.53 | 0.54 | 0.55 | 0.59 | 0.58 | 0.61 | 0.65 | 0.65 | 0.67 | 0.72 | 0.76 | 0.81 | 0.84 |
| Office visits (m) | 12.00 | 12.36 | 12.78 | 13.16 | 14.05 | 13.73 | 13.16 | 12.63 | 13.32 | 13.55 | 14.23 | 14.87 | 15.26 | 15.54 | 16.22 | 17.09 | 17.27 | 18.04 |
| Lab results* (m) | 73.33 | 78.00 | 79.50 | 79.76 | 81.69 | 86.79 | 87.98 | 87.12 | 91.55 | 95.22 | 101.10 | 103.89 | 107.66 | 108.06 | 111.77 | 119.92 | 126.70 | 132.76 |
| Outpatient med dispense (m) | 16.86 | 17.37 | 17.84 | 17.28 | 18.34 | 19.12 | 19.64 | 20.42 | 21.19 | 21.73 | 22.66 | 23.52 | 24.22 | 24.97 | 26.80 | 28.39 | 29.53 | 30.21 |
| Blood pressure assessment** (m) | |  |  |  |  |  |  | 9.93 | 10.93 | 11.19 | 11.57 | 11.91 | 12.03 | 12.26 | 12.80 | 13.38 | 13.72 | 14.14 |
| Body weight assessment** (m) | |  |  |  |  |  |  | 7.42 | 8.39 | 8.66 | 9.31 | 9.68 | 9.74 | 10.14 | 10.71 | 11.19 | 11.34 | 11.65 |
| Prophylactic vaccine (m) | 0.59 | 0.65 | 0.81 | 0.73 | 0.84 | 0.81 | 0.95 | 1.01 | 1.25 | 1.43 | 1.70 | 1.76 | 1.76 | 1.90 | 2.16 | 2.16 | 2.30 | 2.53 |
| Delivery (k) | 25.32 | 25.51 | 25.23 | 23.97 | 23.91 | 24.90 | 25.84 | 26.38 | 26.01 | 26.27 | 28.07 | 29.92 | 30.05 | 30.71 | 32.70 | 34.52 | 34.86 | 35.98 |
| EDG (k) | 26.70 | 28.74 | 28.87 | 26.75 | 27.62 | 27.85 | 29.09 | 42.48 | 45.94 | 48.47 | 49.80 | 53.03 | 53.62 | 55.84 | 59.06 | 68.03 | 71.70 | 74.91 |
| Colonoscopy (k) | 30.04 | 35.77 | 40.10 | 39.10 | 49.60 | 60.12 | 68.26 | 97.80 | 105.92 | 109.70 | 113.44 | 122.7 | 124.22 | 123.77 | 125.59 | 135.35 | 144.81 | 156.12 |
| Sigmoidoscopy (k) | 52.46 | 42.60 | 38.49 | 36.77 | 38.49 | 32.50 | 30.75 | 31.47 | 29.89 | 36.86 | 27.95 | 22.83 | 17.89 | 14.60 | 11.92 | 11.45 | 10.96 | 9.65 |
| FOBT/FIT (k) | 87.76 | 97.50 | 100.65 | 99.70 | 93.84 | 154.23 | 252.79 | 301.70 | 311.23 | 312.73 | 331.29 | 347.22 | 370.52 | 381.65 | 412.21 | 415.13 | 415.01 | 437.72 |
| Cardiac catheterization  /coronary arteriography (k) | 6.99 | 11.74 | 12.61 | 12.07 | 12.59 | 10.63 | 7.49 | 8.96 | 9.62 | 11.45 | 11.34 | 11.03 | 12.23 | 13.47 | 15.15 | 16.10 | 14.20 | 12.14 |
| Electrocardiography (k) | 353.63 | 403.10 | 420.31 | 461.20 | 488.63 | 502.61 | 487.38 | 499.24 | 527.10 | 551.03 | 557.40 | 570.27 | 558.19 | 559.94 | 600.79 | 653.86 | 672.17 | 726.32 |
| Pulmonary function test (k) | 54.08 | 77.63 | 60.58 | 49.93 | 42.73 | 45.06 | 33.34 | 44.86 | 94.19 | 90.54 | 99.35 | 97.64 | 101.77 | 97.16 | 96.96 | 99.16 | 100.90 | 103.25 |
| CT scan** (k) |  | 12.17 | 58.88 | 115.78 | 184.38 | 215.02 | 242.58 | 263.41 | 284.18 | 293.53 | 317.47 | 329.50 | 332.72 | 348.14 | 383.03 | 415.90 | 463.81 | 510.09 |
| MRI scan** (k) |  | 6.09 | 23.50 | 55.75 | 83.88 | 97.89 | 107.22 | 118.93 | 127.74 | 134.11 | 144.85 | 151.57 | 158.22 | 168.94 | 193.68 | 207.04 | 223.75 | 239.58 |
| Ultrasound** (k) |  | 18.58 | 65.08 | 117.39 | 178.56 | 229.14 | 278.39 | 295.13 | 320.97 | 352.72 | 386.46 | 419.52 | 441.90 | 462.43 | 492.84 | 519.93 | 551.50 | 587.26 |
|  | 2001 | 2002 | 2003 | 2004 | 2005 | 2006 | 2007 | 2008 | 2009 | 2010 | 2011 | 2012 | 2013 | 2014 | 2015 | 2016 | 2017 | 2018 |
| ***Crude prevalence****** |  |  |  |  |  |  |  |  |  |  |  |  |  |  |  |  |  |  |
| Hospital admission /kp | 93.85 | 94.76 | 95.42 | 109.24 | 111.23 | 101.01 | 97.28 | 99.55 | 97.50 | 96.75 | 91.22 | 85.31 | 82.36 | 79.04 | 76.68 | 71.29 | 67.64 | 65.75 |
| ER visits /kp | 363.82 | 320.66 | 296.28 | 286.55 | 279.35 | 259.21 | 255.97 | 255.14 | 267.84 | 260.26 | 261.51 | 266.73 | 260.84 | 260.18 | 264.24 | 262.60 | 266.37 | 265.25 |
| Office visits /p | 6.23 | 6.17 | 6.37 | 6.69 | 7.02 | 6.66 | 6.20 | 5.84 | 6.09 | 6.10 | 6.05 | 6.10 | 6.13 | 6.07 | 5.93 | 5.89 | 5.67 | 5.71 |
| Lab results* /p | 38.06 | 38.94 | 39.62 | 40.56 | 40.81 | 42.09 | 41.45 | 40.33 | 41.85 | 42.89 | 43.02 | 42.65 | 43.24 | 42.24 | 40.83 | 41.34 | 41.61 | 42.01 |
| Outpatient med dispense /p | 8.75 | 8.67 | 8.89 | 8.79 | 9.16 | 9.27 | 9.25 | 9.45 | 9.68 | 9.79 | 9.64 | 9.66 | 9.73 | 9.76 | 9.79 | 9.79 | 9.70 | 9.56 |
| Blood pressure assessment** /p | |  |  |  |  |  |  | 4.60 | 5.00 | 5.04 | 4.93 | 4.89 | 4.83 | 4.79 | 4.68 | 4.61 | 4.51 | 4.47 |
| Body weight assessment** /p | |  |  |  |  |  |  | 3.44 | 3.84 | 3.90 | 3.96 | 3.98 | 3.91 | 3.96 | 3.91 | 3.86 | 3.72 | 3.68 |
| Prophylactic vaccine /kp | 307.05 | 325.65 | 405.67 | 371.76 | 420.47 | 394.47 | 448.34 | 465.30 | 569.09 | 644.85 | 721.73 | 722.18 | 706.58 | 743.83 | 790.23 | 745.21 | 755.64 | 801.90 |
| Delivery**** /kp | 50.24 | 49.26 | 49.74 | 49.15 | 49.15 | 50.07 | 50.41 | 50.69 | 49.62 | 50.03 | 49.66 | 50.92 | 50.50 | 50.20 | 49.72 | 49.26 | 47.26 | 46.84 |
| EDG /kp | 13.86 | 14.35 | 14.39 | 13.61 | 13.80 | 13.50 | 13.70 | 19.66 | 21.00 | 21.83 | 21.19 | 21.77 | 21.53 | 21.83 | 21.58 | 23.45 | 23.55 | 23.70 |
| Colonoscopy***** /kp | 30.44 | 34.43 | 38.38 | 37.45 | 48.00 | 58.04 | 66.40 | 91.06 | 96.65 | 98.50 | 97.75 | 103.54 | 102.07 | 97.64 | 92.39 | 94.54 | 96.61 | 99.40 |
| Sigmoidoscopy***** /kp | 59.13 | 44.63 | 39.86 | 38.35 | 40.11 | 32.71 | 30.70 | 29.61 | 26.35 | 30.45 | 21.07 | 15.61 | 10.61 | 7.52 | 4.87 | 3.88 | 3.23 | 2.71 |
| FOBT/FIT***** /kp | 84.48 | 89.03 | 90.17 | 89.97 | 81.55 | 145.79 | 260.86 | 309.26 | 328.40 | 330.60 | 337.92 | 347.42 | 361.56 | 350.55 | 356.83 | 340.81 | 327.23 | 328.42 |
| Cardiac catheterization /coronary arteriography/kp | 3.63 | 5.86 | 6.28 | 6.14 | 6.29 | 5.15 | 3.53 | 4.15 | 4.39 | 5.16 | 4.83 | 4.53 | 4.91 | 5.26 | 5.53 | 5.55 | 4.66 | 3.84 |
| Electrocardiography /kp | 183.53 | 201.27 | 209.48 | 234.54 | 244.10 | 243.71 | 229.61 | 231.09 | 240.92 | 248.19 | 237.21 | 234.14 | 224.17 | 218.87 | 219.47 | 225.40 | 220.75 | 229.80 |
| Pulmonary function test /kp | 28.07 | 38.76 | 30.19 | 25.39 | 21.34 | 21.85 | 15.71 | 20.77 | 43.05 | 40.78 | 42.28 | 40.09 | 40.87 | 37.98 | 35.42 | 34.18 | 33.14 | 32.67 |
| CT scan** /kp |  | 6.07 | 29.34 | 58.88 | 92.10 | 104.26 | 114.28 | 121.93 | 129.89 | 132.21 | 135.10 | 135.29 | 133.62 | 136.08 | 139.92 | 143.37 | 152.32 | 161.39 |
| MRI scan** /kp |  | 3.04 | 11.71 | 28.35 | 41.90 | 47.46 | 50.51 | 55.05 | 58.39 | 60.41 | 61.64 | 62.23 | 63.54 | 66.04 | 70.75 | 71.37 | 73.48 | 75.80 |
| Ultrasound** /kp |  | 9.28 | 32.44 | 59.70 | 89.20 | 111.11 | 131.15 | 136.61 | 146.70 | 158.87 | 164.46 | 172.24 | 177.47 | 180.75 | 180.04 | 179.23 | 181.12 | 185.80 |

*Chemistry and virology labs; **Information for early years is not available or incomplete; ***Crude prevalence was estimated in adults who enrolled in the health plan for at least 11 months within the calendar year. ****Women 15-44 years of age; *****50-75 years of age; EDG: esophagogastroduodenoscopy, also known as upper gastrointestinal endoscopy; FOBT: fecal occult blood test; FIT: fecal immunochemical test; /p: per person; /kp: per 1,000 persons; med: medication.
