# Supplementary material for: Research data warehouse: using electronic health records to conduct population-based observational studies
Source: JAMIA Open. 2023 Jun 21;6(2):ooad039. doi: 10.1093/jamiaopen/ooad039 (PMC10284679; doi:10.1093/jamiaopen/ooad039)
Supplement: ooad039_Supplementary_Data [file ooad039_supplementary_data.zip › Supplemental File 1.docx]

**Supplemental File 1. Two examples of data integration and standardization**

**Claims consolidation**

Services delivered to KPSC members at non-KPSC facilities are sent to KP for reimbursements by filing claims. There can be several detailed claims associated with each service or visit. Much of the information in these claims focus on the financial aspects of the service and are very inconvenient to use for research directly. Therefore, we developed a consolidation process to combine these multiple claims into single encounters that better fit the research needs of investigators.

- Exclude closed out or denied claim records
- Clean up and standardize hospital revenue code and bill type if the value was not missing
- Regenerate place of service (POS) based on hospital revenue codes and type of bill codes for historic year claims prior 1998 due to unreliable POS.
- Assign the encounter type for each POS. For example, POS=21 as inpatient, POs=23 as emergency visit, POS=11 as office visit.
- Consolidate the claims into encounters for each POS according following rules
  - Combine the claims with same start date, such as office visit (POS=11), home care (POs=12), etc.
  - Combine the claims with consecutive start date and stop date, i.e., there is no gap between two claims. For example, emergency visit (POS=23), Inpatient (POS=21), etc.
- Combine the information (such as diagnosis codes and procedure codes) within each claim into corresponding consolidated encounters.
  - Remove duplicate diagnosis codes within each consolidated encounter if there are multiple identical diagnosis codes from different claims.
  - Remove duplicate procedure codes within same consolidated encounter if there are multiple identical procedure codes from different claims with the same procedure date.

**Vital sign cleaning and standardizing process**

Vital signs including weight and height are collected by care providers during patient visits since the HC implementation in 2004. A number of research studies showed that it is possible for these data to contain errors in height and weight as a result of mistakes made when the information was measured and/or entered in the EMR system. In 2007, a joint effort within R&E developed a series of data cleaning processes based on BMI percentiles for children/adolescents and on BMI for adults. The cleaning strategy, originally designed for outpatient data cleaning, was later implemented for the inpatient data. The cleaning process of Adult BMI and children BMI are implemented individually, and summarized briefly below.

- Clean adult heights with out of normal range values
  - Set to missing if height < 4 feet or ≥ 87 inches
  - Clean adults with height numbers between 4 to 7 inclusive.
  - Apply the 8% rule for adults with three or more height measurements
- Clean adult weights with out of normal range values
- Set to missing if weight < 30 pounds or > 1000 pounds
- Clean patients with only weight measurements in the low-weight category
- Clean patients with only weight measurements in the high-weight category
- Apply the 40% rule for adults with three or more records
- Set child height to missing if any of the following conditions is satisfied
  - Age > 6 months and original height <20 cm
  - Age > 1 year old and original height <1 foot
  - Age < 2 years old and original height ≥4 feet
  - Original height >7 feet 3 inches
  - Age > 2 years old and original height percentile is less than 0.5-percentile, and original height is 12 inches below median first percentile height for age (median of range 0.5–1.5 PERC)
  - Age > 2 years old, original height percentile is greater than 99.5-percentile, and original height is 24 inches above median 99th percentile height for age (median of range 89.5–99.5 PERC).
- Set child weight to missing if any of the following conditions is satisfied
- Age >1 year old and original weight <0.5 kg
- Age >2 years old, original weight percentile is less than 0.5-percentile, and original weight < (median of first percentile of weight for age - 0.3*median of 50th percentile of weight for age) and single underweight measurement (not repeated underweight measurements
- Age >2 years old and original weight percentile is greater than 99.5-percentile and original weight ≥ (median of 99th percentile of weight for age + 2*median of 50th percentile of weight for age) and single overweight measurement (not repeated overweight measurements).
- Calculate BMI based on the cleaned height and weight.
