# Supplementary material for: Research data warehouse: using electronic health records to conduct population-based observational studies
Source: JAMIA Open. 2023 Jun 21;6(2):ooad039. doi: 10.1093/jamiaopen/ooad039 (PMC10284679; doi:10.1093/jamiaopen/ooad039)
Supplement: ooad039_Supplementary_Data [file ooad039_supplementary_data.zip › Supplemental File 2.docx]

**Supplemental File 2. Data Quality Monitoring Reports**

The reports typically include frequency, outliers and trends of data. The following screen short is part of the report examining the annual counts of the following measures from 2008 to 2018 (incomplete for 2018): encounters, diagnosis codes, procedure codes, discharge disposition, admission source, level of service (LOS) code, and visit reason for the **last six versions** of the data. For each measure, the annual counts were stratified by data source, care type (Clinic, Hospital, Virtual and Other) and care sub-type (varies by care type, for example, Office Visits and Urgent Care for Clinic, Inpatient and ER to Hospital). In addition to actual counts, we also included the differences in counts (1) between two consecutive versions (labeled as “CHANGE FROM PREVIOUS VERSION”), and (2) between two consecutive years) (labeled as “CHANGE FROM PREVIOUS PERIOD”. A positive number indicates an increase and a negative number indicates a decrease from the previous version or time period. Monthly counts and weekly counts were examined by a similar approach.


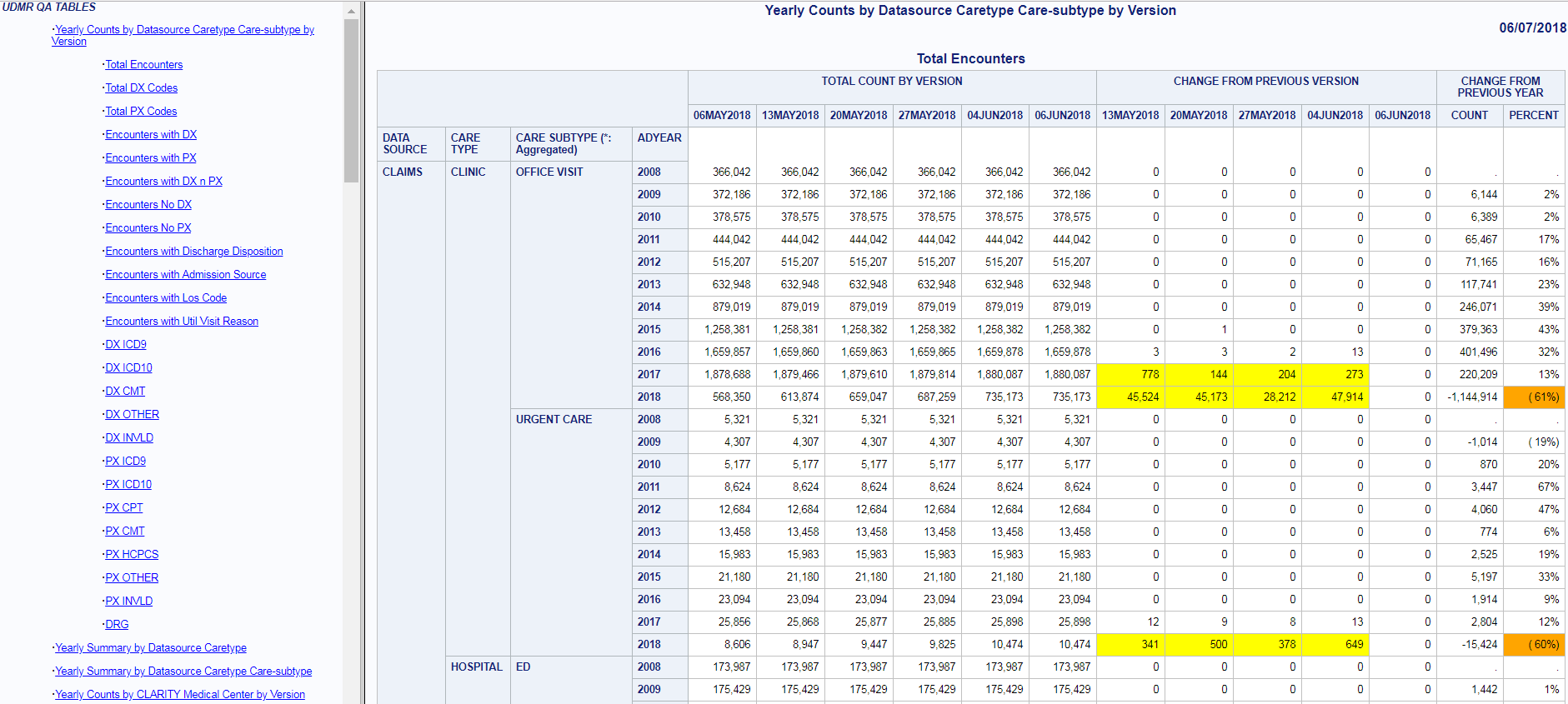


Navigation panel (click to jump to a specific table)

Stratified by data source, care type and care sub-type

Actual counts of the last 6 versions

Difference in counts between two consecutive versions

Difference of Counts and % between two consecutive periods
